# P83 Assessing the reach and engagement of the Antibiotic Guardian Campaign within deprived and minority ethnic groups in England

**DOI:** 10.1093/jacamr/dlag102.089

**Published:** 2026-06-26

**Authors:** Orlagh Quinn, Bee Yean Ng, Ellie L Tang, Diane Ashiru-Oredope

**Affiliations:** UK Health Security Agency, London, UK; UK Health Security Agency, London, UK; UK Health Security Agency, London, UK; UK Health Security Agency, London, UK

## Abstract

**Background:**

The Antibiotic Guardian (AG) campaign was launched in 2014 and encourages healthcare professionals and members of the public to become AGs through an online pledge system. It aims to increase commitment to reducing antimicrobial resistance (AMR), change behaviour, and increase knowledge. AMR burden disproportionally affects minority ethnic and deprived groups. In 2023, updates were made to the pledge process to include demographic data commonly associated with health inequalities. In ESPAUR report 2024 to 2025, ‘Asian or Asian British ethnic group had a resistant bloodstream infection rate that was almost 2 times higher than the White ethnic group (92.5 versus 32.0 cases per 100 000 people).’ ESPAUR also reported that ‘areas with higher levels of deprivation had a greater burden of antibiotic-resistant bloodstream infections, with a 47.2% higher rate of resistant bloodstream infections compared to the lowest level (43.3 versus 29.4 cases per 100 000 people)’.

**Objectives:**

To assess the reach and engagement of the Antibiotic Guardian Campaign within deprived and minority ethnic groups previously identified as disproportionately affected by AMR burden in England.

**Methods:**

Demographic and pledge data were extracted for all Antibiotic Guardians who made a pledge via the main website page between August 2014 – December 2025. R version 4.3.1 was used to describe trends and demographics, including Index of Multiple Deprivation (IMD) quintile, in English AGs using frequencies and percentages on pledges made.

**Results:**

There were 195 793 Antibiotic Guardian pledges made via the main website page between August 2014 and December 2025; of those, 123 639 were made in England, 100 624 by healthcare workers and 10 038 by members of the public. The English Index of Multiple Deprivation quintiles for AGs shows 25% are in 1st quintile (the most deprived areas, *n*=30 820), 24% in 2nd (*n*=29 355), 20% in 3rd (*n*=23 972), 18% in 4th (*n*=21 427) and 13% in the 5th quintile (least deprived areas, *n*=16 245). Since the AG pledge form was updated in March 2023, there have been 27 422 pledges, of which 24 280 are from the England. The most pledges were made by those in the 35-64 (*n*=10 997) and 18-34 (*n*=9767) age groups. Of AGs for whom their ethnicity was collected, 60% were White (*n*=13 200), 25% Asian or Asian British (*n*=5380), 5% Black, African, Caribbean or Black British (*n*=1114), 3% Other (e.g. Arab, *n*=753) and 2% were Mixed ethnicity (*n*=389); 5% selected ‘Prefer not to say’ (*n*=1051).

**Conclusions:**

Demographic data of Antibiotic Guardians highlights high levels of engagement among those working or living in more deprived areas and among those from minority ethnic groups compared to the general population in England: White (86%), Asian or Asian British (8%), Black, African, Caribbean or Black British (3%), Mixed ethnicity (2%) and Other (1%). Future work may wish to explore motivations to become an Antibiotic Guardians among these populations.

